# Views of policy makers and health promotion professionals on factors facilitating implementation and maintenance of interventions and policies promoting physical activity and healthy eating: results of the DEDIPAC project

**DOI:** 10.1186/s12889-017-4929-9

**Published:** 2017-12-06

**Authors:** Saskia Muellmann, Berit Steenbock, Katrien De Cocker, Marieke De Craemer, Catherine Hayes, Miriam P. O’Shea, Karolina Horodyska, Justyna Bell, Aleksandra Luszczynska, Gun Roos, Lars Jørun Langøien, Gro Rugseth, Laura Terragni, Ilse De Bourdeaudhuij, Johannes Brug, Claudia R. Pischke

**Affiliations:** 1 Leibniz Institute for Prevention Research and Epidemiology – BIPS, Bremen, Germany; 2 Department of Movement and Sports Sciences, Ghent University, Ghent, Belgium; 3 Department of Public Health and Primary Care, Institute of Population Health, School of Medicine, Trinity College Dublin, University of Dublin, Dublin, Ireland; 4 Department of Psychology, SWPS University of Social Sciences and Humanities, Wroclaw, Poland; 5 Trauma, Health, & Hazards Center, University College of Applied Sciences Colorado, Colorado Springs, CO USA; 6 Consumption Research Norway – SIFO, Oslo and Akershus, Oslo, Norway; 7 Department for Physical Education, Norwegian School of Sports Science, Oslo, Norway; 8 Oslo and Akershus University College of Applied Sciences, Oslo, Norway; 9 Faculty of Social and Behavioural Sciences, University of Amsterdam, Amsterdam, The Netherlands

**Keywords:** Multi-level intervention, Policy, Physical activity, Diet, Adoption, Implementation, Maintenance, DEDIPAC, Europe, Case studies

## Abstract

**Background:**

The uptake, implementation, and maintenance of effective interventions promoting physical activity (PA) and a healthy diet and the implementation of policies targeting these behaviors are processes not well understood. We aimed to gain a better understanding of what health promotion professionals and policy makers think are important factors facilitating adoption, implementation, and maintenance of multi-level interventions and policies promoting healthy eating and PA in Belgium, Germany, Ireland, Norway, and Poland.

**Methods:**

Six interventions and six policies were identified based on pre-defined criteria. Forty semi-structured interviews were conducted with stakeholders from various sectors to elicit information on factors impacting adoption, implementation, and maintenance of these interventions and policies. All interview transcripts were coded in NVivo, using a common categorization matrix. Coding in the respective countries was done by one researcher and validated by a second researcher.

**Results:**

Active involvement of relevant stakeholders and good communication between coordinating organizations were described as important factors contributing to successful adoption and implementation of both interventions and policies. Additional facilitating factors included sufficient training of staff and tailoring of materials to match needs of various target groups. The respondents indicated that maintenance of implemented interventions/policies depended on whether they were embedded in existing or newly created organizational structures in different settings and whether continued funding was secured.

**Conclusions:**

Despite considerable heterogeneity of interventions and health policies in the five countries, stakeholders across these countries identify similar factors facilitating adoption, implementation, and maintenance of these interventions and policies.

**Electronic supplementary material:**

The online version of this article (10.1186/s12889-017-4929-9) contains supplementary material, which is available to authorized users.

## Background

A healthy diet, regular physical activity (PA), and the reduction of sedentary behavior (SB) are lifestyle factors known to help prevent or delay the onset of non-communicable diseases (NCDs), including cardiovascular diseases and cancers, and to improve overall physical and mental health [1, 2]. The World Health Organization (WHO) launched a global action plan in 2013 comprising six steps to increase the levels of these protective behaviors at the population-level and to further reduce additional behaviors known to increase NCD risk [3]. One of these steps addresses the underlying social determinants of modifiable risk factors for NCDs by suggesting the creation of health promoting environments. Actions to create health promoting environments may include the implementation of effective interventions and policies aimed at promoting a healthy diet and PA and at reducing SB. Such public health and policy interventions may target behavior change by operating at multiple levels, such as the social level by addressing social norms related to health behaviors in interventions, or by changing aspects of the built environment to impact behavior [4–7].

In the ‘Determinants of Diet and Physical Activity’ (DEDIPAC) Knowledge Hub [8], one of the aims was to gain a better insight into factors that facilitate adoption, implementation, and maintenance of multi-level interventions and policies to promote healthy eating, PA (and reduce SB). In a first step, two umbrella reviews were conducted [9, 10]. The first umbrella review was aimed at identifying good practice characteristics of interventions and policies promoting a healthy diet, PA, and the reduction of SB [10]. Using the WHO framework [11], 53 good practice characteristics were identified and subsequently categorized into three domains: (1) main intervention/ policy characteristics, referring to the design, targets, and participants, (2) monitoring and evaluation processes, and (3) implementation issues. The aim of the second umbrella review was to identify evidence-based conditions for successfully implementing interventions and policies promoting a healthy diet, PA, and a reduction in SB [9]. Using the RE-AIM (Reach, Efficacy, Adoption, Implementation and Maintenance) framework [12], 83 potential conditions that were identified in 112 publications (50 systematic reviews, 17 stakeholders’ documents, and 45 position review papers) were grouped into five domains: (1) reach in the target population (*n* = 8; e.g., strategies facilitating recruitment processes, cultural and social issues relevant for reaching target populations), (2) efficacy (n = 8; e.g., satisfaction with implementation, feasibility and acceptability), (3) adoption by the target staff, setting, or institutions (*n* = 24; e.g., community support for implementation, governmental and legislative involvement), (4) consistency, costs, and adaptations made during the implementation process (*n* = 43; e.g., accessibility for participants, cultural context of implementation), and (5) maintenance of the effects in individuals and settings over time (*n* = 3; e.g., sustainability). The vast majority of implementation conditions (73 of 83) were supported by documents referring to both interventions and policies. Only seven policy-specific implementation conditions were identified. For example, increasing complexities of coexisting policies/legal instruments and a lack of politicians’ collaboration in implementation were stated as barriers to policy implementation. Merely three implementation conditions were specified for interventions only: the degree to which an intervention is delivered as intended (compared to the protocol), assessment of fidelity of delivery, and involving available human resources in the delivery. To conclude, results of these two umbrella reviews suggest that the number of research papers on implementation conditions has grown rapidly in recent years.

However, there is still lack of research providing more details on implementation processes from the perspective of health promotion professionals, policy makers, and practitioners working in the field. Glasgow and colleagues [13] noted that many interventions are developed and successfully evaluated but most of them get “lost in translation” because they are not sustainably implemented in the different “real life” settings. To facilitate long-term improvements in PA, sedentary and dietary behaviors, stakeholders in these settings ought to play a major role in the implementation and maintenance of interventions/ policies after completion of research projects. However, little is known about factors facilitating or impeding policy or intervention implementation from the stakeholders’ point of view. Therefore, information collected directly from intervention and policy stakeholders, using qualitative approaches, is important. Hence, the aim of the present article is to explore what health promotion professionals, policy makers, and various other stakeholders think facilitates or impedes the adoption, implementation, and maintenance of interventions/ policies promoting healthy eating, PA, and a reduction in SB.

## Methods

### Aim and setting of the study

For that purpose, six intervention and six policy cases of five different countries were identified based on pre-defined criteria. Subsequently, 40 semi-structured interviews were conducted in Belgium, Germany, Ireland, Norway, and Poland with various stakeholders of these cases. In these interviews, conditions for successfully implementing and maintaining interventions and policies, as well as issues or obstacles encountered during the process of implementing and sustaining these interventions and policies were discussed. Interventions were eligible for inclusion when they were theory-based and multi-level (i.e., they used knowledge of the behavioral determinants at individual, social, and physical environmental levels to improve dietary behavior, PA, and/or SB in individuals). A policy was defined as a purposive and consistent course of action to stimulate a healthy diet and/or PA, formulated by a specific political process, and adopted, implemented, and enforced by a public agency, such as the European Union (EU), EU member states, or regional or local governments. Both, interventions and policies could be research projects or publicly funded/ government funded endorsed strategies.

### Case selection

Potentially relevant interventions and policies were identified via multiple channels varying by country. In Belgium, Ireland, and Poland, brief scoping reviews of publically available literature were performed and followed up with informal meetings with local interventionists/practitioners and researchers. In Germany, a literature search on multi-level interventions/policies promoting a healthy diet, SB, and/or PA was conducted, using national and international databases. In Norway, results from policy evaluation which was performed as part of another subproject of the DEDIPAC project were used to identify cases. An overview of the cases can be found in Table 1.

**Table 1:** Overview of selected cases for qualitative case studies in five DEDIPAC member states investigating conditions for successful implementation and maintenance

General information on intervention/policy					Case selection		
Country	Name of intervention/policy	Short description of intervention/ policy	Target group	Implementation status	Intervention/policy well-described?	Intervention/ policy evidence-based?	Intervention/ policy feasible and transferable?
Belgium	10,000 Steps (intervention) [14–17]	A multi-strategy community-based intervention to promote PA in adults. A local media campaign, a website, environmental approaches, the sale and loan of pedometers, and several local PA projects were concurrently implemented.	Adults aged 18 or older	Currently implemented	Yes	Yes	Yes
Belgium	Tutti Frutti (intervention) [18]	Tutti Frutti is a fruit and vegetables project carried out in Flemish schools. The project aim is that schools choose one or more fixed days per week on which children can eat a piece of fruit or vegetable during the break.	Primary school children (6–12 years old)	Currently implemented	Yes	Yes	Yes
Germany	IDEFICS (intervention) [19, 20]	IDEFICS is a lifestyle intervention (diet, PA, stress reduction) for the prevention of childhood obesity which was implemented in various settings (i.e., kindergarten, schools, communities) and targeted children and (grand)parents, as well as stakeholders in these settings.	Preschool and school children	Completed in 2012	Yes	Yes	Yes
Germany	Federal state offices coordinating networks for the provision of healthy food options in schools (policy) [24]	The ‘Federal state offices coordinating networks for the provision of healthy food options in schools’ supports schools nationwide to develop and improve the quality of a balanced meal selection in schools.	School children	Currently implemented	Yes	Partly	Partly
Ireland	Food Dudes Healthy Eating Programme (intervention) [21]	The aim of this intervention is to increase school children’s consumption of fruits and vegetables at school and at home.	School children	Currently implemented	Yes	Yes	Yes
Ireland	Green Schools Programme Active travel theme (intervention) [22]	The aim of this intervention is to promote sustainable modes of transport to school (e.g., walking, cycling) and carpooling, and public transport use.	School children	Currently implemented	Yes	Partly	Yes
Norway	Keyhole labelling (policy) [25]	The aim of this policy is to make it easier to choose healthier foods.	General population	Currently Implemented	Yes	Yes	Partly
Norway	Free school fruit scheme (policy) [26, 27]	The aim of this policy is to make school children and adolescents consume more fruit and vegetables.	School children and adolescents	Ended in 2014	Yes	Yes	Partly
Poland	European Schools for Healthy Food - Slow Food in the Canteen (intervention) [23]	The intervention promotes the consumption of fresh healthy food in canteens at primary schools.	Primary school children	Currently implemented	Yes	Yes	Yes
Poland	Fit student (policy) [28]	The main objective of this policy is to prevent obesity among children and adolescents by identifying students at risk for obesity.	School children	Currently implemented	Yes	Yes	Yes
Poland	Tasty, Healthy, Valuable (policy) [29]	This policy is aimed toward promoting a healthy diet through advice provided by a municipality-employed specialist (nutrition advisor) who visits the participating schools and provides detailed informational support regarding options for changing the assortment of school shops and by changing the style of cooking in canteens.	Primary school children	Currently implemented	Yes	Yes	Yes
Poland	Fit city (policy) [30]	The main aim of this policy is to form pro-health behaviors in the local community.	General population living in Wroclaw city	Currently implemented	Yes	Yes	Yes

Cases were selected based on criteria which were previously developed in the DEDIPAC-consortium. A consensus document had been developed defining good practices for policies or interventions as (1) well-described, (2) evidence-based, and (3) feasible and transferable. In each of the five involved countries, a minimum of two cases per country was selected. We first aimed to select only cases that met all of the inclusion criteria, using the judgement of the involved researchers in each country. However, it was not feasible to exclude cases that did not fully meet the criteria because this would have limited the representation of several countries in the study. Hence, the selected cases are a convenience sample of interventions/ policies in the participating countries. More detail regarding which cases met all or fewer than three criteria can be found in Table 1. A secondary aim in the search for eligible cases was to select a sufficient number of both policy and intervention cases for both behaviors to be able to provide a balanced picture of the current situation of policy and intervention implementation and factors affecting these processes in Europe.

Interventions and national, local or regional policies were eligible for inclusion if: a) they were completed in the past decade or ongoing, b) targeted diet, PA, or SB or all three behaviors, c) a description of all intervention components and levels, including results of an outcome evaluation or a description of the policy, was available. The final selection of cases was made by the involved researchers in the respective countries and depended on the willingness of representatives of policy/ intervention cases to participate in the study. In total, 12 cases, six interventions and six policies, were selected which are described briefly (by country) in the following section.

### Description of intervention cases

In Belgium, two intervention cases were investigated in the qualitative case studies. The “10,000 Steps” intervention was initiated in Ghent in 2005 and is based on the original “10,000 Steps Rockhampton” project in Australia. “10,000 Steps” is a community-based intervention which is currently implemented in the whole of Flanders (Dutch-speaking part of Belgium) that aims to promote PA in adults [14–17]. “10,000 steps” is designed to intervene at the personal (e.g., pedometer sale/ loan), social (e.g., workplace projects), and environmental (e.g., walking circuits) levels. “Tutti Frutti” is an ongoing Flemish intervention promoting the consumption of fruit in primary school children at schools. “Tutti Frutti” is part of a European school fruit program and has been implemented since 2003. Schools choose a fixed day in the week where all children eat a piece of fruit or vegetable as a snack. In addition, an educational package is offered to schools focusing on increasing children’s knowledge on healthy eating, skills needed to make a healthy choice, and attitudes towards healthy eating [18].

In Germany, one intervention case was selected for analysis. The “Identification and prevention of Dietary- and lifestyle-induced health EFfects In Children and infantS (IDEFICS)”-intervention was a lifestyle intervention (diet, PA, stress reduction) for the prevention of childhood obesity that was implemented in various settings (i.e., kindergarten, schools, communities) and targeted children and (grand)parents, as well as stakeholders in these settings (i.e., kindergarten staff, teachers, local public authorities and influential stakeholders in the community) [19]. “IDEFICS” was implemented in eight European countries between 2006 and 2012. Hence, all intervention materials were centrally developed, culturally adapted, and then used for implementation in the participating countries. The effectiveness of this intervention was evaluated in a large-scale European study, including 16,220 2–10-year-old children in Belgium, Cyprus, Estonia, Germany, Hungary, Italy, Spain, and Sweden [20]. The German “IDEFICS” intervention was implemented in day care centers and primary schools in a city in Northern Germany.

In Ireland, two intervention cases were selected for investigation. The ongoing “Food Dudes Healthy Eating Programme” was developed by researchers in Bangor University in Wales to encourage primary school children to consume more fruits and vegetables by delivering fruits and vegetables directly to the schools on a daily basis for 16 days, followed by recording fruits and vegetables brought to school by children themselves. The “Food Dudes Healthy Eating Programme” was initially piloted in Ireland in two schools in 2002/2003 by Bangor University. An “extended pilot” was carried out in 98 schools between 2005 and 2007. A national rollout began in 2007, funded by the Irish Government until 2009. Since the 2009/2010 academic year, the cost of delivery of the fruits and vegetables for the intervention phase is funded through the EU School Fruit Scheme, while all other expenses are funded nationally [21]. The second intervention case was the “Green Schools Programme”, an ongoing environmental education program, environmental management system and award scheme that promotes and acknowledges long-term, whole school action for the environment. The “Green Schools Programme” has seven themes, one (the Active Travel theme) of which was examined in further detail in the case study. The travel theme was piloted in the Greater Dublin Area between 2005 and 2007 and was rolled out nationally since 2008. The aim was to promote sustainable modes of transport (e.g., walking, cycling) and carpooling, and public transport use [22].

In Poland, one intervention case was examined in a qualitative case study. The “European Schools for Healthy Food – Slow Food in the Canteen” intervention promoted the consumption of fresh and healthy foods in primary school canteens. The intervention lasted for 1 year (2011); however, since then it was incorporated into the schools’ curricula and is continued by involved teachers [23]. The “European Schools for Healthy Food – Slow Food in the Canteen” intervention used knowledge of the behavioral determinants at different levels (individual, social, and environmental) to improve dietary behavior among students. The aims were to educate children about a healthy diet during daily classes, to create opportunities to involve parents in intervention activities, and to rebuild school canteens to improve the quality of the served food.

### Description of policy cases

In Germany, one policy case was selected for analysis. The “Federal state offices coordinating networks for the provision of healthy food options in schools”-policy is part of a national initiative to promote a healthy diet and PA in Germany (i.e., Germany’s national initiative to promote healthy diets and physical activity (INFORM)). The overall aim of this policy is to improve the quality of school catering. For policy implementation, 16 federal state offices were established between 2009 and 2010 (one in each of the 16 German federal states). Policy funding was secured by the Federal Ministry of Food and Agriculture until the end of 2016, since then policy implementation is funded by the 16 federal states [24].

In Norway, two policy cases were selected for investigation. The “Keyhole” label which was established in Sweden in 1989 became a common Nordic symbol labelling system in Denmark, Sweden, and Norway in 2009. The “Keyhole” is a voluntary food label, promoted and controlled by Public Authorities that currently identifies healthier (less fat, sugar and salt, and more dietary fibre) food products within different product groups [25]. In 2003, a nationwide school fruit subscription program for grades 1–10 was established. In fall 2007, the “Free school fruit program” was implemented in all Norwegian lower secondary schools (grades 8–10) and all combined primary and secondary schools (grades 1–10). In 2008, this policy was passed as a law [26, 27]. However, when the political power shifted in Parliament in 2014, funding for this program was ended and the law was abolished.

In Poland, three policy cases were selected for the qualitative analysis. All of them are still currently implemented. The policy “Fit Student” is a city-based program which was implemented in educational institutions in Wroclaw and aimed to prevent obesity among children and adolescents by identifying students at risk for obesity. Weight and height of students participating in the program were measured. Afterwards, the parents of the students with a high body mass index received a letter with information about the results and a recommendation for consultations with four specialists: an endocrinologist, a nutritionist, a psychologist and a physiotherapist. The so called “4 door consultations” were free of charge. The pilot of the program was implemented in 2011. At the end of 2014, 68 primary schools had participated in the program. The program is still ongoing [28]. The policy “Tasty, Healthy, Valuable” was initiated in 2014 and aimed to promote a healthy diet through advice provided by a municipality-employed specialist visiting the participating schools and providing detailed information on options for changing the assortment of school shops and the style of cooking in canteens. Currently, 68 kindergartens and 131 schools in Wroclaw participate in the program [29]. The policy “Fit City” is a city-based program in Wroclaw aiming to foster health promoting behaviors in the local community. “Fit City” offers a range of free of charge educational activities referring to PA (e.g., workshops, PA classes, Wroclaw’s Health-Promotion Days, PA week). The policy has been implemented since 2003 [30].

### Interview participants

The overall aim was to interview stakeholders from at least two of the following stakeholder groups: national, local, regional government; local communities (i.e., community leaders); national, local, regional organizations (e.g., youth, elderly, sports clubs, charity societies, working groups); social services and welfare sector (e.g., kindergartens, schools, retirement homes); agencies and companies in the private sector (e.g., food companies); health care providers; physicians; research (i.e., scientists, study coordinators); developers of interventions/ policies (e.g., researchers, representatives of foundations or health insurances); persons working in the individual settings who were involved in implementing an intervention/ policy (e.g., school staff). Table 2 shows the number of interviews conducted by stakeholder group. The selection of cases and interviewees was conducted by partners in each country. Potential cases were identified in a literature search on interventions/ policies promoting a healthy diet and PA in national or international databases (i.e., in Germany), following a brief scoping review of publically available literature and informal meetings with local interventionists/practitioners and researchers (i.e., in Belgium, Ireland, Poland), or among the potential candidates of the DEDIPAC database with good practices and results from policy evaluation (i.e., Norway). After selecting intervention/ policy cases, partners in each country searched for relevant stakeholders for the selected intervention/ policy (e.g., search for contact details of intervention/ policy owner/ implementer, asking already contacted intervention/ policy owner/ implementer for additional potential interviewees). Potential interviewees were contacted via e-mail. E-mails included a cover letter, the study information, and objectives. Potential interviewees were informed that participation in the case study was based on informed and freely given consent and could be revoked at any time. All interviewees were of full legal age. Study participation was not incentivized.

**Table 2:** Number of interviews by stakeholder group

Case study	Interviewees				
	Project coordination	Implementer (e.g., school staff)	Government	Other stakeholder	Total
Belgium					
*Intervention:* 10,000 steps	2	0	0	0	2
*Intervention:* Tutti Frutti	1	0	1	0	2
Germany					
*Intervention:* IDEFICS	1	2	0	1	4
*Policy:* Federal state offices coordinating networks for the provision of healthy food options in schools	0	3	0	0	3
Ireland					
*Intervention:* Food Dudes Healthy Eating Programme	2	3	1	1	7
*Intervention:* Green Schools Programme - Travel theme	3	3	1	1	8
Norway					
*Policy:* Keyhole labelling	2	0	0	1	3
*Policy:* Free school fruit	1	0	1	1	3
Poland					
*Intervention:* European Schools for Healthy Food - Slow Food in the Canteen	0	2	0	0	2
*Policy:* Fit Student	2	0	0	0	2
*Policy:* Tasty, Healthy, Valuable	1	1	0	0	2
*Policy:* Fit City	1	1	0	0	2
Total	15	15	7	3	40

### Data collection

The data collection took place between December 2014 and April 2015 by means of face-to-face or telephone interviews in the respective national languages (Dutch, English, German, Norwegian, and Polish). Interviews were audio recorded and transcribed, using transcription software (i.e., NVivo, f4). The interviews lasted 20–70 min (ranges, Belgium: 33–58 min, Germany: 20–47 min, Ireland: 28–69 min, Norway: 30–60 min, Poland: 35–70 min).

### Interview guides

Based on the conditions for implementation and maintenance of interventions and policies identified in the second umbrella review described in the introduction [9], two qualitative semi-structured interview guides, one for intervention and one for policy cases, were developed. The interview guides contained open questions regarding factors (1) which facilitated or (2) hindered successful implementation; (3) strategies to overcome barriers and to (4) boost sustainability after completion of the implementation (see Additional file 1). Moreover, interview guides contained specific prompts to elicit further information in case the open questions did not yield sufficient information. In the subsequent analysis of the data, these prompts were categorized into three groups reflecting three phases in the implementation of an intervention/ policy: adoption, implementation, and maintenance.

### Data analysis

The involved researchers analysed the data which were collected in the individual case studies following the approach for content analysis by Elo and Kyngäs [31] and the instructions for data analysis which were provided by the coordinating team in Germany. All involved researchers coded their interview transcripts in NVivo, using a common categorization matrix. Coding in the respective countries was done by one researcher and validated by a second researcher. Data were grouped and summarised into themes, using Microsoft Excel. Again, one researcher reduced the data and a second researcher checked whether their analysis would result in the same or a similar reduction of the data. Any discrepancies in the coding or reduction process were discussed until consensus between researchers was reached. National summary reports were drafted and sent to the coordinating team for an overarching analysis of all data, using the same approach for coding and reduction described above. Lastly, two researchers from the coordinating team selected quotes illustrating the results of the overarching analysis.

## Results

The results section is structured as follows: Firstly, results from the interviews conducted for the intervention cases are reported followed by those gathered in the policy cases. For each category of the respective interview guides, a brief summary of results across countries is provided. Exemplary quotes of stakeholders’ comments in these countries can be found in Tables 3 and 4. Furthermore, results of the case studies are organized to reflect the three phases of implementation described above.

**Table 3:** Quotes for intervention cases

Facilitating factors	
Adoption	
Staff expertise for implementation	“.. all of those decisions were very practically driven and very much…what will work on the ground, what do we do know from our experience, [name] is a former teacher as well so the two of us can be really advocating for teachers and explaining how schools operate and what would…and knowing what would work and what would not.” [Ireland, Food Dudes Healthy Eating Programme]
Training for implementation	“… of course, we trained the employees in regard to the different modules and the structure of the intervention, as well as, certainly, regarding goals and main objectives, or target group. All of this was practiced in a four-day-workshop at the time, if I remember it correctly.” [Germany, IDEFICS]
Communication and collaboration	“Within the Flemish Government, we have a very good collaboration for this project, which is a positive thing. […] Within the Government, we work together with different policy domains, but we also have private organisations such as VIGEZ (Flemish expertise center for health promotion and disease prevention) that are connected with the department of health, but actually it is a private organization.” [Belgium, Tutti Frutti] “…now some local authorities are brilliant, like the Road Safety Officers would have their job in schools and talk about road safety and they’ve really teamed up with us, they go into our schools and talk about road safety.” [Ireland, Food Dudes Healthy Eating Programme]
Implementation	
Delivery characteristics: dose & fidelity	“This means that activities or measures which had been implemented in the settings were scrutinized for completeness and that [the documentation forms] were completed together with all participants of each monthly round table meeting in order to be able to keep close track of the processes and the implementation of activities and to determine afterwards what had been implemented and what had already been part of the curriculum.” [Germany, IDEFICS]
Adjustments and customizations	“…with special schools it’s been very much about working with the teachers in those schools to apply the principles behind the programme, em but to match them to the needs of the children and their specific need. So even within a class in a special school there might be different children who are being rewarded for doing different things…for some if they have a terrible aversion to em to bananas or yellow foods, then just to even have the banana in the same room as them might be a huge (prompt) step forward and it’s about edging them closer and closer to eating.” [Ireland, Food Dudes Healthy Eating Programme] “They have the freedom to use their own logo, house style, communication strategies and even another intervention ‘name’.” [Belgium, 10,000 steps] “We designed a system whereby the rewards would be packaged and labelled per phase and per day. So phase 1 box would arrive…it could be packed per classroom as well. So the teacher would open the box for his or her own classroom inside that would be all the phase 1 rewards, clearly packaged and labelled day 1 reward, day 2 reward, day 3 and so forth for the 16 days and that they would have if they had 28 in their class, they’d have 28 pencils or 28 sharpeners or they’d have …the number predefined. Em and then the same for phase 2 that everything was clearly labelled.” [Ireland, Food Dudes Healthy Eating Programme] “We have a few children that maybe just wouldn’t kind of like fruit and veg at all. And they actually did try it, because they really wanted the prize”. [Ireland, Food Dudes Healthy Eating Programme]
Characteristics of the setting affecting delivery/ implementation	“I think engagement of both principals and teachers is important.” [Belgium, Tutti Frutti] “… what settings we address, what setting is probably not quite appropriate. Keywords here are e.g.: child day care centers which had a different pedagogical approach, e.g., day care centers for children with speech problems, Waldorf or Montessori kindergartens. These are day care centers which could only to a small extent identify with our concept. The selection and the tips, of course, were given to us in the round table meetings because representatives from these settings were already present.” [Germany, IDEFICS] “Internal attitude, this inner motivation, the awareness of the importance of the programme (...) You have to catch the bug yourself, even just taking the lifestyle.” [Poland, European Schools for Healthy Food – Slow Food in the Canteen]
Implementation process evaluation	“Yes, there have been 2 evaluations already. One in 2006 and 2007. So before it became a European story. That was a process evaluation and effect evaluation.” [Belgium, Tutti Frutti] “We have a monitoring survey every 3 years in which the following is checked: how is education for physical activity taken care of? What type of methods do you adopt? What are your facilitating factors? For 10,000 Steps, we also ask about the adoption rate and the degree of anchoring.” [Belgium, 10,000 steps]
Maintenance	
Dissemination	“We keep on informing the schools. Every school year, we send a newsletter to the schools with information about the project. This is also available on our website. We have the Facebook-page which gives some ludic information, some nice recipes, some activities that are being carried out, or some schools who organize activities and send us the pictures of these activities. In this way, we try to pass on as much information as possible.” [Belgium, Tutti Frutti] “We managed to organize a conference, where we invited representatives from the local government. It was also attended by health and safety representatives (…) we prepared a recording of it for the TV station.” [Poland, European Schools for Healthy Food – Slow Food in the Canteen]
Obstacles	
Adoption	
Communication and collaboration	“Often, only the sport services are implementing ‘10,000 Steps’, while the other sectors are even not aware of this. So this means that a part of the evidence-based character of the intervention is not fulfilled, as there should be strategies in all contexts in which physical activity can occur: home, work, leisure and transport. Without communication between the sectors, not all contexts are being targeted properly.” [Belgium, 10,000 steps]
Implementation	
Adjustments and customizations	“… the accompanying materials were in some cases considered not as very fitting [the needs of the target group] by some colleagues.” [Germany, IDEFICS] “It is not the case that we have ready-to-use materials for each and every single group: ‘OK, you work with this group? Here you have this to use’, it is not like that.” [Belgium, 10,000 steps]
Accessibility and time issues	“It might have been wrongly judged, as the e-portal is not very easy for everyone, especially not for schools.” [Belgium, Tutti Frutti] “We have lost members of our round table meetings because the strict requirements for documentation were so time-consuming that some lost their enthusiasm at some point or another.” [Germany, IDEFICS] “We are an infant school so we have a limited number of hours every day in an already overloaded curriculum so. That was the concern that we had going into it.” [Ireland, Food Dudes Healthy Eating Programme]
Cultural context	“…it’s not the boys are more expendable but they are expected to do slightly dangerous things. Girls are expected not to. They are expected to be on the pink bike going around the park with their pals not out on the road.” [Ireland, Green Schools Programme - Travel theme] “... changes to the norms or family values, that is a big challenge in DEIS (disadvantaged) schools, if you give people fruit and veg in a school and then they go home and their mammies give them a batter burger and chips.” [Ireland, Food Dudes Healthy Eating Programme]
Costs and funding/ resources needed for delivery	“In small municipalities there is for example no health promotion department and therefore a lack of time and manpower to implement the intervention. Also the crisis can cause a lack of funding.” [Belgium, 10,000 steps] “The reach of the intervention decreases when there is no guaranty that the consumer will have step counters. This is the biggest threat for the intervention, especially in disadvantage groups, as there is often a need for external funding to implement step counters”. [Belgium, 10,000 steps]
Characteristics of the settings affecting delivery/ implementation	“A switch in personnel can be ‘deadly’. If someone implementing the intervention is leaving, a lot of knowledge and networking contacts can be lost if a new staff member is not oriented soon.” [Belgium, 10,000 steps] “The internal organization of the schools, which is also a problem. That is something that we have known for a while now. But we cannot do anything about it with the Flemish Government.” [Belgium, Tutti Frutti] “Particularly academic stakeholders, such as teachers, are always more difficult. Kindergarten educators, for example, a large group, proved much more cooperative than persons higher in the social hierarchy such as teachers, who were very discerning to the point that some of them left altogether.” [Germany, IDEFICS] “If we had more money we could have a proper kitchen (...) the lady [the cook] has to finish preparing lunch and so they [students] have to meet up in the afternoon or early in the morning so that they are not interrupting their normal work. They would need a place for themselves, with equipment so that they could cook for themselves.” [Poland, European Schools for Healthy Food – Slow Food in the Canteen] “…not just in the rural environment, in some urban schools too…children have to travel right out of their estates along a main road to the school as opposed to having permeability through their local estates.” [Ireland, Green Schools Programme - Travel theme] “…because this is quite a new school, em there was em difficulties with getting the pathways and the roadways finished outside. And so everything inside the school was done, pathways and cycle lanes and cycle huts and stuff, but it was outside on the road here. So it was difficult for us to be promoting something until that really had changed.” [Ireland, Green Schools Programme - Travel theme]

**Table 4:** Quotes for policy cases

Facilitating factors	
Adoption	
Training for implementation	“We organized individual meetings with each group of implementers. We also conducted a content and organizational training. In terms of the implementation of the programme, we showed what the programme should look like in terms of what we require. We had further training delivered by specialists from the Medical University and the University of Life Sciences.” [Poland, Fit Student]
Adoption in physical environment	“The fact that there was a type of subscription scheme maybe also was a factor that supported the implementation of free school fruit.” [Norway, Free school fruit program] “This programme functions well because there is a range of other already-existing programmes that accompany it, e.g. ‘The Health-promoting Schools’.” [Poland, Fit Student] “It is like this, in [name of city] we served as vanguard for the expansion of full-time schools in Germany. In the course of the expansion the issue of school meals entered the discussion at an early stage. The idea of implementing a central point of contact for these issues came up even before this federal and federal state project.” [Germany, Federal state offices coordinating networks for the provision of healthy food options in schools]
Governmental and legal involvement	“In the elections 2005 in the campaign some focus on school meals. One party wanted free hot school meals and two other parties wanted a free school meal. When the three parties formed a coalition I think free school fruit came. Because they didn’t get free school meals they had to come with something.” [Norway, Free school fruit program] “The development and expansion of full-time schools in the federal state is, of course, a big influential component.” [Germany, Federal state offices coordinating networks for the provision of healthy food options in schools]
Collaboration and communication	“Ähm, clearly, it is beneficial that we are not primarily ecotrophologists. We have one colleague and she comes from ecotrophology, another one is a teacher and I am originally a teacher and a trained cook myself. This combination helps us getting perceived as partners by kitchen staff, caterers, and meal service providers.” [Germany, Federal state offices coordinating networks for the provision of healthy food options in schools] “The topic ‘school meals’ is so multi-layered that it is necessary to cooperate with other professions and other partners or else you will not make any progress.” [Germany, Federal state offices coordinating networks for the provision of healthy food options in schools] “We invited representatives of the Medical University, the University of Natural Sciences, health-promoters, school nurses and behavioural nurses, nutritionists. Together we designed the programme and planned activities. So there was a multi-sectoral collaboration: scientific, academic, educational and it all helped in developing a more realistic delivery of the programme.” [Poland, Fit Student]
Implementation	
Delivery characteristics	“... when the Nordic countries meet there is a certain instinct for competition, so it’s like If they can do it, so can we.” [Norway, Keyhole] “The participants who regularly took part in the workshop received different prizes from our partners: tickets to sport halls, cultural and pro-health places as well as some gadgets. We notice that the mere fact of rewarding is very important (...) it is a good motivational system for them.” [Poland, Fit City]
Simplicity of the policy	“For both the industry and the government, because we knew that the Keyhole, sort of, worked more or less in Sweden. It was 20 years old or something.” [Norway, Keyhole] “We know from the last consumer research that one in two people say that the labelling makes it easier to choose healthy [products].” [Norway, Keyhole]
Costs and funding/ resources needed for delivery	“I think it had not been this successful had we not had long term funding of 5 years then and now three additional years. This is crucial in attempting to motivate people to cooperate and to commit themselves to this kind of quality. Long-term funding definitely was a good thing.” [Germany, Federal state offices coordinating networks for the provision of healthy food options in schools] “We did our [evaluation] with such [consumer] inquiries. Lots of questions on what it means and so on ... But in other ways ...? Yes, they follow the product development, I think.” [Norway, Keyhole]
Maintenance	
Long-term funding and political support	“There has been control campaigns with the other Nordic countries, and now it is a part of the control [made by The Norwegian Food Safety Authority] ... It is necessary that we [The Norwegian food Safety Authority] prioritise the labelling.” [Norway, Keyhole] “From the Federation we get the message that the VNS is the most successful of the entire INFORM campaign and that there are several ministers stating that – depending on election cycles – they would like to pursue this project in some fashion.” [Germany, Federal state offices coordinating networks for the provision of healthy food options in schools]
Obstacles	
Adoption	
Adoption in physical environment	“The fact that the education system is public and not private [was the obstacle] because it meant that the financing was very limited. Wanting to equip the kitchen, the management of a particular [educational] institution will not ask the parents for the money to get a new oven, they can only inform the parents about it.” [Poland, Tasty, Healthy, Valuable] “There is no room in the canteen, the capacities are not sufficient. Plus, it is political, too. When children wish to seize a full-time school program and the head of school has to admit that they do not have the capacities. Or if you find out that children having been accepted into full-time school programs have to eat their meals in the classrooms.” [Germany, Federal state offices coordinating networks for the provision of healthy food options in schools]
Governmental and legal involvement/ collaboration and communication	“Regulations - we had to get EU’s approval, and that took three to 4 months. But that was probably not the worst part. The worst part was reaching an agreement between Norway and Sweden on the criteria.” [Norway, Keyhole] “And that is, yeah, yeah, I said it in the beginning, the range of consultations offered is very broad. But there are quite a lot of people, many stakeholders who have their word in this, as well, and who have the authority to make decisions in other places.” [Germany, Federal state offices coordinating networks for the provision of healthy food options in schools] “Of course, the success of the VNS [Federal state offices coordinating networks for the provision of healthy food options in schools] or the gaining of sponsorship/financial carriers depends on the political atmosphere in the federal state. Considering [name of city], where one financial carrier is very committed, the impact and significance of the VNS is quite different. This is one factor sometimes impeding work in some federal states.” [Germany, Federal state offices coordinating networks for the provision of healthy food options in schools] “To me it was a little bit puzzling that it was so little thought through the implementation and that it was not planned more collaboration and involvement from all partners.” [Norway, Free school fruit program]
Community use	“In schools there are no contact persons for this topic, and too few people are responsible. This makes it difficult to address the proper persons who may be able to put something into action.” [Germany, Federal state offices coordinating networks for the provision of healthy food options in schools] “After 5 years, we indeed have schools where neither the school principal nor the parents have an interest in the topic.” [Germany, Federal state offices coordinating networks for the provision of healthy food options in schools]
Implementation	
Costs and funding/ resources needed for delivery	“The amount allocated by a ministry, particularly at the federal state level, does not increase. One is somehow dependent on good will from the ministries and that they will put money into this project and not into another measure.” [Germany, Federal state offices coordinating networks for the provision of healthy food options in schools] “There were really some misunderstandings. Some municipalities believed that they didn’t get funding anymore. They thought that they had to have free school fruit but that they didn’t get any money from the State anymore. They misunderstood.” [Norway, Free school fruit program] “With three full time positions in charge of 3300 schools we are unable to provide counselling to every single school.” [Germany, Federal state offices coordinating networks for the provision of healthy food options in schools]
Implementation process evaluation	“There are no financial means for any type of evaluation. Of course, evaluation of our work is mandatory. We keep documentation of our counselling activities and events. It is possible for participants to evaluate our events, but these are issues that we were left alone with.” [Germany, Federal state offices coordinating networks for the provision of healthy food options in schools]
Other factors	“... no real opportunity to sanction, other than making complaints and then you were on the blacklist and there were some in the media.” [Norway, Free school fruit program]

### Results of the intervention case studies

#### Facilitating factors concerning the adoption of interventions

The expertise of the implementation staff, stemming from previous experiences in and knowledge of the school environment, contributed to a successful implementation of interventions implemented in schools (for quotes see Table 3 facilitating factors - staff expertise for implementation). In addition, training of the implementation staff in aims, sequence, and delivery of intervention activities at the beginning and during the implementation of the intervention was revealed as a facilitating factor during the adoption phase (for quotes see Table 3 facilitating factors - training for implementation). Furthermore, interviewees described the involvement of relevant stakeholders, inter-sectoral collaboration, and good communication between the coordinating organization and the government, private organizations and settings as important factors contributing to a successful adoption of interventions (for quotes see Table 3 facilitating factors - communication and collaboration).

#### Facilitating factors concerning the implementation of interventions

Implementation was facilitated if implementers delivered the intervention according to a protocol to ensure standardized delivery. Interviewees also thought that a detailed documentation of intervention activities was necessary to ensure intervention fidelity during implementation (e.g., a monthly documentation) (for quotes see Table 3 facilitating factors - delivery characteristics: dose and fidelity). Adjustments and customizations to tailor interventions to different target groups (e.g., older adults, children with special needs) were also deemed as facilitating factors. Other adjustments during intervention delivery that were raised by interviewees included taking organizational factors of the different settings into account when organizing intervention activities (e.g., school holidays) or allowing implementers to make their own adaptations to intervention materials to match their individual preferences. Furthermore, interviewees stated that simple ready-to-use intervention materials for implementers (and participants), as well as incentives to encourage children to participate were key components of successful delivery mechanisms (for quotes see Table 3 facilitating factors - adjustments and customization). A process evaluation to ensure that possible adjustments could be made to the intervention after the initial roll-out was deemed important. Four interventions were accompanied by process evaluations (Belgium, Ireland, Germany, and Poland) (for quotes see Table 3 facilitating factors - implementations process evaluation).

In regard to characteristics of a given setting influencing implementation, interviewees suggested following the advice from stakeholders regarding the selection of settings for intervention implementation prior to the start of an intervention to ease implementation at a later point in time. When asked which implementers’ characteristics affected intervention implementation, interviewees across countries noted that engagement and motivation of intervention implementers (e.g., school principals, teachers in the school setting) helped successfully implementing given interventions (for quotes see Table 3 facilitating factors - characteristics of the setting affecting delivery/implementation).

#### Facilitating factors concerning the maintenance of interventions

Training for implementers in the settings and days with special intervention themes and activities facilitated maintenance of interventions. Information for maintaining interventions was spread through local authorities, newsletters, press conferences, intervention websites, and social media (e.g., Facebook; for quotes see Table 3 facilitating factors - dissemination).

#### Obstacles to the adoption, implementation, and maintenance of interventions

Several conditions that hindered an effective adoption and implementation of interventions were pointed out by interviewees. In regard to adoption, limited involvement and support of relevant stakeholders (e.g., from politics, the health sector, parents) and poor communication between involved stakeholders were identified as obstacles (for quotes see Table 3 obstacles - communication and collaboration). Across countries, the issue of intervention materials not being sufficiently tailored to the needs of the target groups was raised as an implementation issue (for quotes see Table 3 obstacles - adjustments and customization). The accessibility of an intervention was in some cases limited by complicated administrative procedures. For example, in one case, schools had to apply for funding at an electronic portal to participate in an intervention. In another case, potential intervention participants could only participate in an intervention if they owned special equipment (e.g., step counters). Other barriers to implementation found to be relevant across countries comprised intervention activities that were too time-consuming or too difficult to integrate into existing curricula or that required lengthy documentation as part of a study (for quotes see Table 3 obstacles – accessibility and time issues).

Changing existing socio-cultural norms regarding eating habits and PA were described as challenges when implementing interventions. In Ireland, for example, cycling was perceived as too risky and unladylike for girls (for quotes see Table 3 obstacles – cultural context). Furthermore, lack of funding for paying implementation staff, as well as for providing intervention materials, such as step counters, were brought up as barriers by interviewees (for quotes see Table 3 obstacles – costs and funding/resources needed for delivery). Finally, interviewees described different characteristics of the settings as impeding implementation of interventions. These included insufficient staff or changes in staff, lack of canteens and/or canteen space, lack of infrastructure supporting walking near rural schools (e.g., no footpaths), curricular commitments of stakeholders (e.g., teachers), and the lack of organizational capacity to organize the delivery of fruit from fruit suppliers (for quotes see Table 3 obstacles – characteristics of the setting affecting delivery/ implementation). No common obstacles to maintaining interventions in all participating countries were identified. Therefore, no exemplary quotes are included in Table 3.

### Results of the policy case studies

#### Facilitating factors concerning the adoption of policies

Interviewees stated that the adoption of policies was often based on political decisions. For example, the “Free school fruit program” was implemented in Norway as long as the party supporting the policy was still in power. In all countries, the involvement of relevant stakeholders from politics and the health and education sectors and of implementers in policy development (e.g., development of materials, pilot testing) and implementation (e.g., information about content of the policy, training for implementers), and a continued collaboration and communication with stakeholders after policy implementation were identified as facilitating factors. Additionally, the adoption of policies was in some cases expedited by structural changes in a given setting. For example, in Germany, an expansion of full-time schools with canteens helped promote healthy school meals (for quotes see Table 4 facilitating factors – governmental and legal involvement). Furthermore, an interdisciplinary team implementing a given policy was considered helpful as it raised the acceptance among the involved stakeholders (for quotes see Table 4 facilitating factors – collaboration and communication).

According to interviewees, municipalities and settings that received information about the content of the policies and how they should be implemented were able to more quickly adopt a given policy. In addition, interviewees recommended training for policy implementation (e.g., in workshops) (for quotes see Table 4 facilitating factors – training for implementation). Furthermore, according to interviewees, the implementation of the examined policies benefited from previously implemented projects or policies that had been similar in content (for quotes see Table 4 facilitating factors – adoption in physical environment).

#### Facilitating factors concerning the implementation of policies

According to interviewees, different strategies were employed to increase motivation to implement a policy in different settings (e.g., a motivational point card system for participants or free participation). In initiatives with several partners, internal competition was regarded as a facilitator for policy implementation. For example, a competition between Nordic countries arose in being best at implementing the “Keyhole” labelling (for quotes see Table 4 facilitating factors – delivery characteristics). The following conditions contributed to the simplicity of the implementation process for the “Keyhole” labelling in Norway: a system for school fruit subscription already existed, the “Keyhole” labelling had already been implemented in Sweden, and it could easily be understood (for quotes see Table 4 facilitating factors – simplicity of the policy).

Long-term funding was considered essential in all countries for ensuring the success of the policy implementation. In addition, some interviewees brought up the importance of policy evaluation as a factor contributing to a successful implementation (for quotes see Table 4 facilitating factors – costs and funding/resources needed for delivery).

#### Facilitating factors concerning the maintenance of policies

Factors contributing to the maintenance of a policy were the availability of long-term funding and political support (for quotes see Table 4 facilitating factors – long-term funding and political support).

#### Obstacles to the adoption of policies

Limited time and spatial capacity at schools (e.g., no facilities to conduct cooking workshops, lack of canteens and staff) hindered policy adoption. One interviewee described that funding problems in the public education system (e.g., for building new canteens or proper equipment of canteens) posed a threat to policy adoption (for quotes see Table 4 obstacles – adoption in physical environment). Furthermore, legal regulations sometimes determined the form of policy adoption. For example, EU approval for the Keyhole label took 3 to 4 months. In addition and prior to approval, Norway and Sweden had to agree on the criteria for the Keyhole label.

Moreover, interviewees pointed out that usually many stakeholders were involved in the process of policy adoption and that the majority of stakeholders had the authority to make decisions with regard to adoption which made it difficult to reach consensus (e.g., in schools, in politics). In addition, it appeared difficult to establish co-operations between the industry and Non-governmental Organizations (NGOs), the education sector, implementers, parents, and the media to increase overall support for policy adoption (for quotes see Table 4 obstacles – governmental and legal involvement/ collaboration and communication). One factor that hindered adoption of the examined policies in the school setting was the lack of expertise or professionals with sufficient skills in schools who could serve as liaisons for the implementation of a nutrition policy. Also, school staff, parents, and authorities had little interest in the topic of the policy (for quotes see Table 4 obstacles – community use).

#### Obstacles to policy implementation

According to interviewees across countries, the implementation of a given policy was affected by available financial resources. Some interviewees perceived the staffing for policy implementation to be limited (for quotes see Table 4 obstacles – costs and funding/ resources needed for delivery). In one case, a lack of information regarding available funding for policy implementation hindered implementation.

A lack of policy evaluation to improve future implementation of policies was raised as an issue. In Germany, financial means for a process evaluation of the policy (under examination in this study) were not allocated by the Federation in neither of the two funding periods. At the federal state level, funds were allocated to evaluate the acceptance of informational workshops among stakeholders. The general goal was to accomplish the evaluation internally in a cost-effective manner by providing bi-annual reports or by documenting counselling activities and events of the “Federal state offices coordinating networks for the provision of healthy food options in schools”. Interviewees reporting on this policy case pointed out that this form of evaluation only generated information about the quantity of activities and none about their quality. In contrast, detailed process evaluations for the “Keyhole” and the “Free school fruit program” were funded in Norway (for quotes see Table 4 obstacles – implementations process evaluation).

Interestingly, concerning one policy case, the lack of enforcement of legal sanctions was raised by interviewees as a barrier. Specifically, in Norway, the implementation of the “Free school fruit program” was difficult because not all municipalities and schools were interested in making adjustments necessary for the implementation of the policy. Some municipalities did not implement the program although it was mandated by law but there was no real possibility to sanction municipalities for not implementing the policy. One option, however; mentioned by interviewees was for parents to complain to the County Governor (the chief representative of King and Government in the county who works for the implementation of central government decisions) who could then send a letter to the municipality (for quotes see Table 4 obstacles – other factors).

#### Obstacles to policy maintenance

Obstacles in regard to maintaining a given policy were too heterogeneous to summarize results across participating countries. No common obstacles could be identified. Therefore, no exemplary quotes are included in Table 4.

## Discussion

The results of 40 interviews conducted with stakeholders of 12 intervention and policy cases in five countries suggest five main factors facilitating intervention/ policy implementation and maintenance: a.) the development and/or existence of stakeholder networks supporting and working towards an implementation of an intervention/ policy, b.) newly created or existing structures in settings to support intervention/ policy implementation, c.) continued funding and political support in the respective country or state, d.) standardized training for staff following detailed intervention/ policy implementation protocols, and e.) socio-cultural adaptations or tailoring of intervention/ policy content to fit the needs and context of the targeted population. The majority of factors contributing to a successful implementation were deemed relevant for both intervention and policy cases across countries. Also, whether an intervention or policy was targeted towards dietary changes or changes in PA made little difference in the issues raised by interviewees.

The results obtained in our study resemble findings reported by Mikkelsen and colleagues [32] who outlined commonalities vs. differences in challenges faced during the implementation of three multi-level multi-component interventions conducted in Europe, North America, and the South Pacific promoting healthy eating and PA to reduce childhood obesity. Similar to our findings, the main challenges identified by the authors included the development of stakeholder networks before and during implementation to continuously support the implementation process, the planning of the dose and intensity needed to reach the target behaviors in various populations (including training and education of staff in the settings), and capacity building to warrant sustainability of interventions after research examining the effectiveness of the intervention is completed. Another important issue raised by Mikkelsen and colleagues [32] which was not mentioned by interviewees in our case studies was the harmonization of delivery mechanisms of the different intervention components across intervention levels or settings (e.g., optimization of the timing of different intervention activities, building activities in later stages of the intervention onto those in earlier stages).

In our study, we also looked at factors influencing policy implementation and found that conditions impacting policy uptake, implementation, and maintenance appeared to be similar to those affecting implementation processes of multi-level interventions to promote healthy eating and PA across participating countries (for an overview of commonalities vs. differences, see Table 5). A recent study, taking a multiple case study approach similar to the one we employed in our study, investigated factors impacting uptake and implementation of a U.S. Department of Agriculture snack policy in schools across several U.S. states [33]. In this study, interviews were conducted with stakeholders from nine high schools across eight states to examine perceptions of school snack implementation. Similar to our study, factors facilitating policy implementation included strong internal and external partnerships with other stakeholders and the incorporation of activities mandated by the policy into existing nutrition education programs.

**Table 5:** Interventions and policies: comparison of facilitating factors and barriers^a^

	Facilitating factors		Barriers	
	Interventions	Policies	Interventions	Policies
Adoption				
Training for implementation	✓	✓	–	–
Staff expertise for implementation^b^	✓	–	–	–
Adoption in physical environment^c^	–	✓	✓	✓
Governmental and legal involvement^c^	–	✓	–	✓
Community use	–	–	–	✓
Collaboration and communication	✓	✓	✓	–
Implementation				
Theory use^b^	–	–	–	–
Delivery characteristics: dose and fidelity	✓	✓	–	–
Adjustments and customizations	✓	-	✓	–
Simplicity	–	✓	–	–
Accessibility	–	–	✓	–
Time issues	–	–	✓	–
Cultural context	–	–	✓	–
Costs and funding/resources needed for delivery	–	✓	✓	✓
Characteristics of the setting affecting delivery/implementation^b^	✓	–	✓	–
Implementers’ characteristics affecting implementation^b^	–	–	–	–
Implementations process evaluation	✓	–	–	✓
Other factors	–	–	–	✓
Maintenance				
Dissemination	✓	–	–	–
Staff and stakeholders: Training for transfer^b^	–	–	–	–
Differences in healthcare systems across countries^b^	–	–	–	–
Long-term funding and political support^c^	–	✓	–	–

^a^Results in the table summarize the main results

^b^Only for interventions

^c^Only for policies

The synthesis of results across countries generated in the DEDIPAC project is currently made available at a designated website (https://www.dedipac.eu/toolbox/) which combines new theoretical frameworks to use as a basis for the development of future interventions/ policies [34], current empirical evidence regarding the effectiveness of multi-level interventions and policies in promoting changes in diet, PA, and SB (i.e., findings of studies and systematic literature reviews), and knowledge of conditions necessary to successfully implement interventions/ policies from a stakeholder’s point of view (i.e., findings of systematic literature reviews and qualitative research). The aim of the website is to make this evidence available to researchers and practitioners in the field of health promotion implementing current interventions or planning new interventions and to assist policy makers in shaping future health policies in the respective countries. Because of the heterogeneity in funding situations, systems (e.g., education systems), and political situations in the five countries, country-specific results will be reported by researchers involved in this study which will allow for interpretations of the data and specific recommendations for future intervention/ policy development in light of the socio-cultural context of each country.

A few examples of unique barriers/ facilitators which were reported in single countries are the following. For example, in Ireland, poor road and transport infrastructure (e.g., a lack of pathways and cycle lanes outside of schools) impeded the implementation of a program promoting active travel of students from and to schools. In Germany, stakeholders who had been involved in the implementation of an intervention to promote healthy eating and PA stated that the research institution in charge of implementation had not sufficiently integrated the program activities into existing school curricula. As a result, stakeholders at times felt that day-to-day activities at schools were interrupted or disturbed by the required intervention activities. In Norway, positive consequences of the implementation of the “Keyhole” food label to promote healthy food options were described by stakeholders. As a result of almost 10 years of policy implementation, there has been an increase in the number of products with the label, as well as items purchased by consumers. Reasons for this development highlighted by interviewees were that the “Keyhole” focuses on basic products that are bought by many; that it is now a common Nordic labelling which means that it has a more secure footing; and thirdly that there is a regular dialogue between the government and the industry with a common interest in sustaining the label. Because of that the implementation of the “Keyhole” continues to be well prepared and there is continuous dissemination.

Overall, the qualitative research approach appeared well suited to obtain an overview of conditions facilitating or hindering uptake, implementation, and maintenance of interventions and policies promoting a healthy diet and PA. Nonetheless, employing other qualitative methods, such as fieldwork and observations, may have provided additional information. Furthermore, the generalizability of the results is limited because the case studies were conducted in selected countries not representing the whole of Europe. In addition, our findings may not be generalizable across other interventions or policies. Several methodological limitations regarding the selection of policy and intervention cases and of interviewees were noted. Firstly, the classification of cases for the different countries as either intervention or policy was difficult because often no clear distinction between intervention and policy was made in practice. However, investigators in all countries used the definitions from the original grant proposal of the DEDIPAC study to categorize a case as a policy/ intervention. Secondly, it was not possible to realize the original aim of the study which was to include an equal number of successful, as well as unsuccessful or less successful cases, meaning that the final selection of cases consisted of more cases which were rather successful at implementing and maintaining interventions/ policies. Thirdly, the sample of interviewees was a convenience sample. Willingness to participate in the case studies seemed to be somewhat higher when interviewees were convinced of the effectiveness of the intervention/ policy and when they personally had made positive experiences while implementing an intervention/ policy. Similarly, social desirability may have played a role during interviews because interviewees may have felt more comfortable reporting facilitating than hindering implementation conditions. Both, data collection in a convenience sample of cases and interviewees and social desirability, may have caused a bias in the data gathered in the case studies towards reporting of more positive results, rather than an accurate reflection of the reality of the implementation process. Lastly, a recall bias may have affected responses of interviewees because in some cases the intervention/ policy had been implemented five to 10 years prior to the case studies and interviewees had trouble accurately remembering the implementation phase of a given intervention/ policy. For example, in Germany, the “IDEFICS” intervention had been implemented more than 5 years prior to the interviews.

Also, interview prompts derived from the systematic umbrella literature review on conditions for intervention/ policy uptake, implementation, and maintenance/ transferability [9] may have limited the responses of interviewees. These prompts were used to elicit new information from interviewees on important conditions for the implementation of interventions and policies to alter dietary behavior and/or PA in “real-life” settings. However, our results indicate that most of the conditions that were identified as relevant for implementing interventions and polices in the literature review were also raised by the interviewees in the case studies. Beyond the conditions that were prompted in the interviews, factors highlighted as relevant by interviewees included the consideration of daily routines in settings during intervention/ policy implementation, raising awareness regarding policy/ intervention themes (e.g., nutrition guidelines) in a given setting, environmental conditions, such as poor road and infrastructure affecting policy implementation, and the importance of an international exchange among policy stakeholders of different countries to facilitate learning from experiences made in other countries.

## Conclusions

To conclude, five main factors (i.e., development and/or existence of stakeholder networks, newly created or existing structures in settings to support intervention/ policy implementation, continued funding and political support, standardized training for staff, and socio-cultural adaptations or tailoring of intervention/ policy content) facilitating intervention/ policy implementation and maintenance were identified in this study which appeared to be similar across the five involved European countries. Recommendations for both policy and practice derived from these findings include building stakeholder networks in favor of and continuously supporting a given policy or intervention and using or adding onto existing structures in a given system or setting to successfully implement and maintain future policies and interventions. To ensure a standardized implementation across settings or intervention levels and possibly across countries, detailed implementation protocols should be drafted and followed. Funding and political situations, as well as socio-cultural context, require adaptations of intervention/policy content to reach target populations in different countries.

This project is one of the first to provide a stakeholder-informed overview of conditions necessary for effectively implementing interventions and policies targeted towards changing diet and PA at the population-level. The results contribute to a better understanding of factors impacting on adoption, implementation, and maintenance of existing interventions and policies in Europe comparing these processes in different countries. They may also inform intervention/policy stakeholders and policy makers’ decisive actions with regard to future implementation of such interventions/ policies.

## Additional file

Additional file 1: Interview guides. (DOCX 54 kb)

## Abbreviations

DEDIPAC: Determinants of Diet and Physical Activity Knowledge Hub
EU: European Union
IDEFICS: Identification and prevention of Dietary- and lifestyle-induced health EFfects In Children and infantS
INFORM: Germany’s national initiative to promote healthy diets and physical activity
NCDs: Non-communicable diseases
NGOs: Non-governmental Organizations
PA: Physical activity
RE-AIM: Reach, Efficacy, Adoption, Implementation and Maintenance
SB: Sedentary behavior
WHO: World Health Organization
