# Supplementary material for: Views of policy makers and health promotion professionals on factors facilitating implementation and maintenance of interventions and policies promoting physical activity and healthy eating: results of the DEDIPAC project
Source: BMC Public Health. 2017 Dec 6;17:932. doi: 10.1186/s12889-017-4929-9 (PMC5718005; doi:10.1186/s12889-017-4929-9)
Supplement: Additional file 1: — Interview guides. (DOCX 54 kb) [file 12889_2017_4929_MOESM1_ESM.docx]

**Additional file 1:**

**1.1 Interview guide: Intervention cases**

**Open questions**

- “From your point of view, which factors contributed to a successful implementation of the [XYZ] intervention?”
- “Could you please describe these factors in further detail?”
- “From your point of view, which factors hindered/ inhibited/ slowed down the implementation of the [XYZ] intervention?”
- “Could you please describe these factors in further detail?”
- “From your point of view, how can these factors/ issues be addressed? How can possible barriers be overcome?”
- “From your point of view, what made this specific intervention sustainable after the implementation phase was completed? If it was not sustained, what were the reasons for the lack of sustainability?”

**Prompts**

If the interviewee does not provide information regarding all factors listed below, you may use the prompts to elicit further information. Please keep in mind that you do not have to ask all of these questions. You may ask about the topics that have not been addressed in response to the open questions stated above. Please do not read out the headings in square brackets to the interviewee.

| **Prompts** | **Already discussed** | **Prompted** |
| --- | --- | --- |
| **1. ADOPTION** |  |  |
| **[Training for implementation]**  “Was the implementation staff trained sufficiently for the adoption of the intervention (e.g., certificate training, workshops, training instructions, regular meetings/ supervision, technical assistance)?” | ⭘ | ⭘ |
| **[Staff expertise for implementation]**  “Do you think that the program staff’s skills, knowledge and competence were adequate to implement the program?” | ⭘ | ⭘ |
| **[Community use]**  “Was local community involvement secured (e.g. use of community organizations, relationships/ networks between implementing organizations and community organizations)?” | ⭘ | ⭘ |
| **[Collaboration and communication]**  “Were multiple stakeholders involved in the program implementation (stakeholders from health and sport sectors, also food, transportation, planning and housing, green spaces, education, healthcare, social services)? How did implementers and stakeholders communicate? Which methods were used to facilitate the communication process?” | ⭘ | ⭘ |
| **2. IMPLEMENTATION** |  |  |
| **[Theory use]**  “Was implementation theory used to facilitate the implementation of the intervention (e.g., the RE-AIM model)? If yes, explain how? From your point of view, did the use of theory contribute to a successful implementation? If yes, how? If not, why not? If implementation theory was not used, could you please explain why this was not done?” | ⭘ | ⭘ |
| **[Delivery characteristics: dose & fidelity]**  “Was intervention dose and fidelity monitored with an implementation protocol? In other words, did you monitor how much of the intervention was delivered and how close the intervention activities were to the intervention protocol? If yes, how was this protocol used? From your point of view, did the monitoring contribute to a successful implementation? If yes, how? If not, why not? If no protocol was used, what was done instead to ensure intervention fidelity?” | ⭘ | ⭘ |
| **[Adjustments and customizations]**  “Was the intervention tailored to  a.) the target population (e.g., socio-cultural background, gender, participants’ needs),  b.) local conditions or the setting,  c.) expertise of the staff responsible for implementing the intervention.  If any of the options are answered with a yes: Please describe in further detail how the intervention was tailored? Could you please explain what worked well with regards to the tailoring? What worked less well?” | ⭘ | ⭘ |
| **[Simplicity of the intervention]**  “From your point of view, was the intervention easy to follow? Were the components easy to understand? If yes, how did the simplicity of the intervention facilitate participation? If not, please elaborate how a lack of simplicity may have affected implementation?” | ⭘ | ⭘ |
| **[Accessibility]**  “Was the intervention accessible to participants? Did the implementation conditions facilitate/ hinder the implementation (e.g., Was participation in the intervention free or if not, was participation affordable? Did the physical environment support the intervention goals, e.g., if an intervention goal was regular walking, a feature of the physical environment enhancing this behavior could be the availability of footpaths, stairs)? If yes, how? If not, why not?” | ⭘ | ⭘ |
| **[Time issues]**  “Were there time issues affecting the implementation of the intervention? If yes, could you describe in detail which issues affected the implementation and how (e.g., lack of time in the community involved/among intervention staff to implement intervention/among intervention participants)? If there were no issues, could tell us about the time schedule for the implementation?” | ⭘ | ⭘ |
| **[Cultural context]**  “From your point of view, did the intervention address specific cultural characteristics of the target population? If yes, how? If not, why not?” | ⭘ | ⭘ |
| **[Costs and funding/ resources needed for delivery]**  “Was sufficient funding provided for the implementation and maintenance of the intervention (in terms of staff, materials)? If yes, could you please give more details on the funding? If not, why was it insufficient? Which problems occurred?” | ⭘ | ⭘ |
| **[Characteristics of the setting affecting delivery/ implementation]**  “Were there any characteristics of the setting that affected the intervention implementation? If yes, which ones, and how did they affect the implementation (e.g., organizational practices/ culture, policies)?” | ⭘ | ⭘ |
| **[Implementers’ characteristics affecting implementation]**  “How did characteristics of the implementers affect the implementation (e.g., expectations/ motivation/ perceived control regarding the process of the implementation)?” | ⭘ | ⭘ |
| **[Implementations process evaluation]**  “Was a process evaluation conducted? If yes, which factors were assessed and documented, and how long was the follow-up period? If not, please explain why not?” | ⭘ | ⭘ |
| **3. MAINTENANCE** |  |  |
| **[Factors contributing to sustainability]**  “Was there an institutionalization of the program (e.g. integration into existing institutional programs)? If yes, how was it integrated? If not, why not? Which strategies were used to promote long-term participation? Which of them were more successful and which ones were less successful?” | ⭘ | ⭘ |
| **4. TRANSFER** | | |
| **[Dissemination]**  “Which actions were taken to disseminate the program? Which channels were used for dissemination? (e.g., media, informal channels)? What worked/ did not work?” | ⭘ | ⭘ |
| **[Staff and stakeholders: Training for transfer]**  “Was the implementation staff trained in the cultural adaptation of such programs?” | ⭘ | ⭘ |
| **[Differences in health care systems across countries]**  “If the program was disseminated in various countries, were differences in health care systems taken into account?” | ⭘ | ⭘ |

**1.2 Interview guide: Policy cases**

**Open questions**

- “From your point of view, which factors contributed to a successful implementation of the [XYZ] policy?”
- “Could you please describe these factors in further detail?”
- “From your point of view, which factors hindered/ inhibited/ slowed down the implementation of the [XYZ] policy?”
- “Could you please describe these factors in further detail?”
- “From your point of view, how can these factors/ issues be addressed? How can possible barriers be overcome?”
- “From your point of view, what made this specific policy sustainable after the implementation phase was completed? If it was not sustained, what were the reasons for the lack of sustainability?”

**Prompts**

If the interviewee does not provide information regarding all factors listed below, you may use the prompts to elicit further information. Please keep in mind that you do not have to ask all of these questions. You may ask about the topics that have not been addressed in response to the open questions stated above. Please do not read out the headings in square brackets to the interviewee.

| **Prompts** | **Already discussed** | **Prompted** |
| --- | --- | --- |
| **1. ADOPTION** |  |  |
| **[Training for implementation]**  “Were policy implementers trained sufficiently (e.g., certificate training, workshops, training instructions, skill development)?” | ⭘ | ⭘ |
| **[Adoption in physical environment]**  “Did the existing physical environment support implementation and maintenance of the policy? If yes, how? If no, which environmental factors inhibited the implementation of the policy?” | ⭘ | ⭘ |
| **[Governmental and legal involvement]**  “What was the legal basis of the policy? What was the involvement of the local government/ local politicians in the implementation of the policy?” | ⭘ | ⭘ |
| **[Collaboration and communication]**  “Were multiple stakeholders involved in the policy implementation (stakeholders from health and sport sectors, political stakeholders, also food, transportation, planning and housing, green spaces, education, healthcare, social services)? How did policy implementers and stakeholders communicate? Which methods were used to facilitate the communication process?” | ⭘ | ⭘ |
| **[Community use]**  “Was local community involvement secured (e.g. use of community organizations, relationships/ networks between implementing organizations and community organizations)?” | ⭘ | ⭘ |
| **2. IMPLEMENTATION** |  |  |
| **[Delivery characteristics]**  “How was the delivery of the policy coordinated (e.g., plans for coordination, communication, specific steps, specific settings, inclusion of mass media, professional groups, lay health advisors, and users in the delivery)? If there was no coordination of the delivery, please explain why not?” | ⭘ | ⭘ |
| **[Simplicity of the policy]**  “From your point of view, was the policy easy to implement/ follow? If not, please elaborate how a lack of simplicity may have affected implementation? Was the complexity of existing structures/ systems, coexisting policies and their interrelations as a possible barrier to the implementation of policy [X] considered?” | ⭘ | ⭘ |
| **[Adjustments and customizations]**  “Was the policy tailored to  a.) the target population (e.g., socio-cultural background, gender, participants’ needs),  b.) local conditions or the setting/ community,  If any of the options are answered with a yes: Please describe in further detail how the policy was tailored? Could you please explain what worked well? What worked less well?” | ⭘ | ⭘ |
| **[Accessibility]**  “Did the policy include financial incentives for the promotion of healthy behavior (e.g., financial incentives to sell healthy products cheaper, policies which facilitate lower costs for using indoor physical activity facilities)? If yes, how? If not, why not?” | ⭘ | ⭘ |
| **[Time issues]**  “Were there time issues affecting the implementation of the policy? If yes, could you please describe in detail which issues affected the implementation and how (e.g., lack of time in the community involved/ among staff to implement policy)? If there were no issues, could you explain the timetable for the implementation of the policy?” | ⭘ | ⭘ |
| **[Cultural context]**  “From your point of view, did the policy address specific cultural aspects/ characteristics of the target population? If yes, how? If not, why not?” | ⭘ | ⭘ |
| **[Costs and funding/ resources needed for delivery]**  “Was sufficient funding provided for the implementation and maintenance of the policy? If yes, could you please give more details on the funding? If not, why was it insufficient? Which problems occurred?” | ⭘ | ⭘ |
| **[Implementations process evaluation]**  “Was a process evaluation conducted? If yes, which factors were assessed and documented, and how long was the follow-up period? If not, please explain why this was not done?” | ⭘ | ⭘ |
| **3. MAINTENANCE** |  |  |
| **[Factors contributing to sustainability]**  “Was there a capacity building plan to implement the policy over the long-term (e.g., provision of training/support in organizations)?” | ⭘ | ⭘ |
| **4. TRANSFER** |  |  |
| **[Dissemination]**  “Which actions were taken to disseminate the policy? Which channels were used for dissemination? (e.g., media)? What worked/ did not work?” | ⭘ | ⭘ |
